# Effects of Shoreline Dynamics on Saltmarsh Vegetation

**DOI:** 10.1371/journal.pone.0159814

**Published:** 2016-07-21

**Authors:** Shailesh Sharma, Joshua Goff, Ryan M. Moody, Ashley McDonald, Dorothy Byron, Kenneth L. Heck, Sean P. Powers, Carl Ferraro, Just Cebrian

**Affiliations:** 1 Department of Marine Sciences, University of South Alabama, Mobile, Alabama, United States of America; 2 Dauphin Island Sea Lab, Dauphin Island, Alabama, United States of America; 3 State Lands Division Coastal Section, Alabama Department of Conservation and Natural Resources, Spanish Fort, Alabama, United States of America; Griffith University, AUSTRALIA

## Abstract

We evaluated the impact of shoreline dynamics on fringing vegetation density at mid- and low-marsh elevations at a high-energy site in the northern Gulf of Mexico. Particularly, we selected eight unprotected shoreline stretches (75 m each) at a historically eroding site and measured their inter-annual lateral movement rate using the DSAS method for three consecutive years. We observed high inter-annual variability of shoreline movement within the selected stretches. Specifically, shorelines retrograded (eroded) in year 1 and year 3, whereas, in year 2, shorelines advanced seaward. Despite shoreline advancement in year 2, an overall net erosion was recorded during the survey period. Additionally, vegetation density generally declined at both elevations during the survey period; however, probably due to their immediate proximity with lateral erosion agents (e.g., waves, currents), marsh grasses at low-elevation exhibited abrupt reduction in density, more so than grasses at mid elevation. Finally, contrary to our hypothesis, despite shoreline advancement, vegetation density did not increase correspondingly in year 2 probably due to a lag in response from biota. More studies in other coastal systems may advance our knowledge of marsh edge systems; however, we consider our results could be beneficial to resource managers in preparing protection plans for coastal wetlands against chronic stressors such as lateral erosion.

## Introduction

Salt marshes—positioned at the interface between terrestrial and marine environment—are highly dynamic and ecologically important systems. Chronic disturbances in adjacent land and sea, however, may degrade the quality of ecosystem services they provide. Shoreline erosion is one of the chronic pervasive disturbances, which has resulted in the loss of marsh meadows, specifically in coastal areas experiencing medium- to high-wave energy i.e., the areas exposed to strong, steady, zonal winds and fronts with high wave energies unprotected by shallow offshore topography [1, 2]. Ecologically, marsh loss is a particular concern because once converted to mudflats, marshlands release previously stored sediment, carbon, and nutrients, thus instigating coastal degradation, which often leads to unforeseen economic implications [3, 4]. Lateral erosion and subsequent marsh loss results in diminished habitat values and wave-buffering capacity as well as lowered carbon sequestering ability of coastal areas [5, 6].

Constant biophysical interactions among (1) vegetation dynamics, (2) hydrodynamic forcing (i.e., tidal currents and waves), and (3) sediment availability determine the marsh establishment and lateral expansion on tidal flats. These three factors interact through positive and negative feedbacks, and show a complex interdependency [7–10]. Indeed, a strong capacity of coastal marshes in attenuating tidal currents and waves results in sediment accretion, and consequently increases sediment elevation. Sediment elevation increase, in turn, aids plant growth by reducing inundation period and associated anoxic conditions. Although vegetation canopies may assist in sediment accumulation by attenuating waves, locally this may divert incoming flows to adjacent areas and enhance biomechanical stress, thus limiting effective growth and recruitment of new marsh plants [11]. Feedback mechanisms of biophysical processes are purely density dependent; therefore, the outcome of interactions vary in marshes with different densities [12]. In this regard, fringing vegetation density could play much more important role in marsh expansion and resilience enhancement than originally recognized.

For mid- to long-term temporal scales, many coastal marshes display a cyclic *advance-and-recession* pattern. A period of marsh formation and lateral expansion (*advance*) is followed by a large-scale destruction (*recession*) mainly due to lateral disturbances, thus leaving marsh edges vulnerable to erosion [13–15]. A major storm surge usually initiates a cascade of processes, namely, sediment destabilization, marsh cliff/escarpment formation, severe erosion along the edges, and eventual marsh collapse [15]. Generally, due to wave-induced slumping, shorelines experiencing high energy are characterized by steep escarpments, whereas, low-energy areas display gentle-sloping shorelines along the Gulf coasts [16]. Thus, coastal marshes respond differently to lateral disturbances based on associated amount of energy and local geomorphology. In high-energy areas, marshes may display cliff erosion (loss of the sediment and above- and below-ground compartments) due to energy-laden waves, whereas, in low-energy areas, marshes may undergo population thinning (i.e., vegetation density reduction) due to constant biomechanical stress and sediment removal, thus limiting new plant recruitment [11].

We are cognizant of various biophysical processes and their respective feedbacks in shaping saltmarsh structures in medium- to long-term temporal scale (i.e., 5–20 years). For example, disturbances such as erosion and sediment loss, coastal squeezing, intense eutrophication and relative sea level rise may cause marsh decimation. On the contrary, sediment accretion and mineral inputs, rapid colonization by pioneer species and floral adaptations may help marshes thrive. Specifically, processes such as tides, waves, inorganic mineral supplies and autochthonous organic matter production collectively determine marsh evolution at a particular location, which in turn is reflected in the structural characteristics of saltmarsh flora [1, 6–10]. Despite the importance of fringing vegetation in counteracting lateral disturbances, there remains a considerable gap in empirical evidence about the impact of shoreline dynamics on fringing vegetation in a short-term scale (i.e., 2–3 years). Moreover, the mechanisms by which lateral erosion—a kind of chronic disturbance—affects vegetation density is not fully understood. Knowing this important and outstanding issue may be crucial to resource managers in designing effective protection measures for coastal saltmarshes from this particular stressor. Through this study, we intend to explore the effects of shoreline dynamics (shoreline movement) on vegetation density at two different marsh elevations (i.e., low- and mid-elevations) for a short time interval (i.e., 2–3 years). Specifically, we measured changes in inter-annual shoreline positions of eight 75-meters-long shoreline stretches with varying levels of erosion or advancement along the eroding shores of coastal Alabama, and tested the hypothesis that shorelines with sediment accretion would show increased *Spartina alterniflora* density during the accretion period due to increased sediment elevation. In contrast, we expected decreased *S*. *alterniflora* vegetation density along eroded shorelines due to the loss of the marsh sediment and additional stress to the vegetation. We established permanent quadrats along selected shoreline stretches to determine smooth cordgrass (*Spartina alterniflora*) density; however, during the experiment, some permanent quadrats were lost owing to marsh collapse or scarp erosion (i.e., loss of whole marsh mass) and a few quadrats showed waning density eventually leading to zero values due to population thinning. In our analysis, we did not include the eroded quadrats that were lost due to collapse or cliff erosion; however, quadrats that underwent gradual density decline reaching zero values were included in our analyses.

### Ethics Statement

All necessary permits for field sampling were obtained from the Alabama Department of Conservation and Natural Resources.

## Materials and Methods

### Study Site

**Fig 1:**
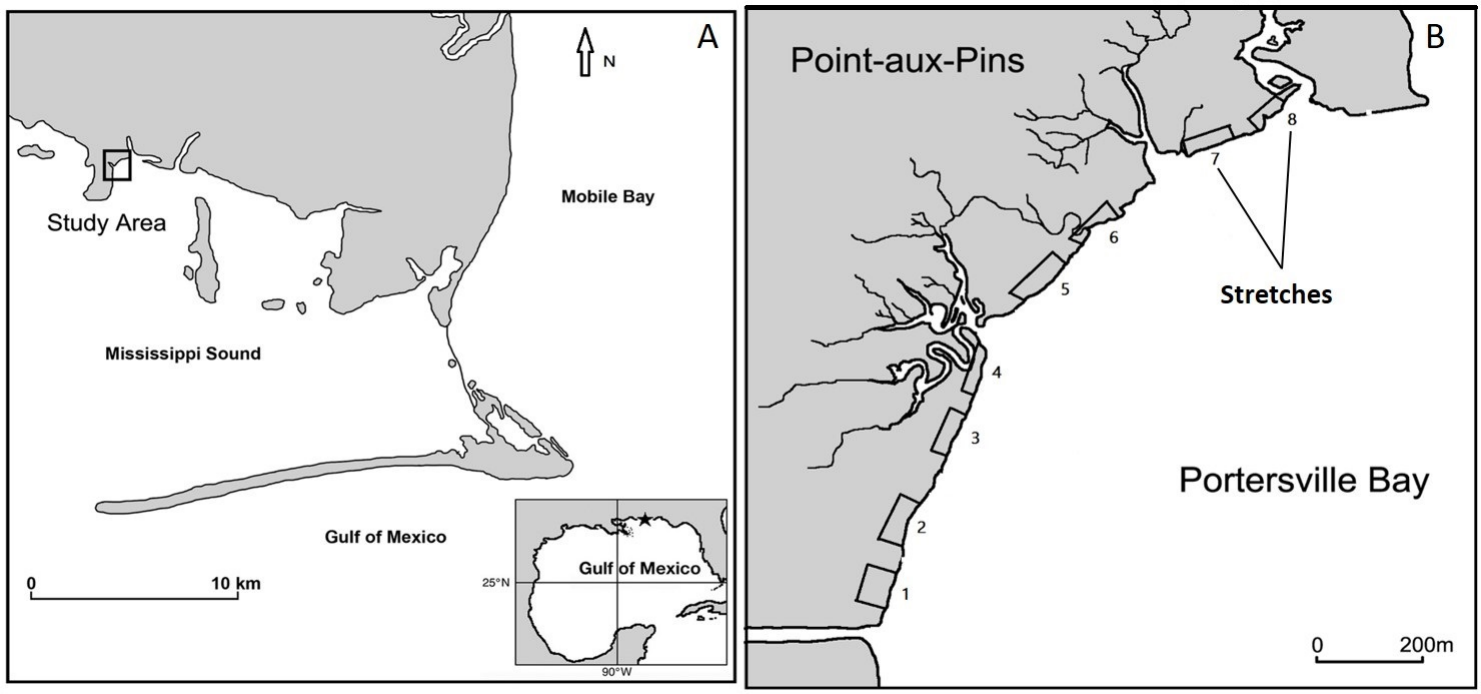
(A) Study site in the Mississippi Sound (B) Surveyed marsh stretches (numbered 1–8) along the shoreline.

Our study site was located in coastal Alabama along historically degrading shores of the northeast portion of a peninsula locally known as North-east Point-aux-Pins (NEPAP; 30 km west of Mobile Bay; site center point 30.383501 N, 88.300453 W; Fig 1A). Tides are diurnal with mean tidal amplitude less than 0.5 m. Dominant winds come from the south/southeast in spring and summer, and cold fronts move in from the north in winter. Monthly wind speed averages ca. 18 km hr^-1^, but large seasonal variability occurs. The fringing marsh edge typically consists of a band of smooth cordgrass (*Spartina alterniflora*) with occasional escarpments especially along the northern ridges. The upper mid- and high-elevation marsh is composed of *Distichlis spicata*, *Borrichia frutescens*, *Batis maritima* and *Spartina patens* [16]. Salinity ranges within 20–27 ppt; although intense rainfall events may drop it to 2 ppt. Mean water-column suspended solid concentration oscillates between 71–97 mg L^-1^ [17].

### Sampling

**Fig 2:**
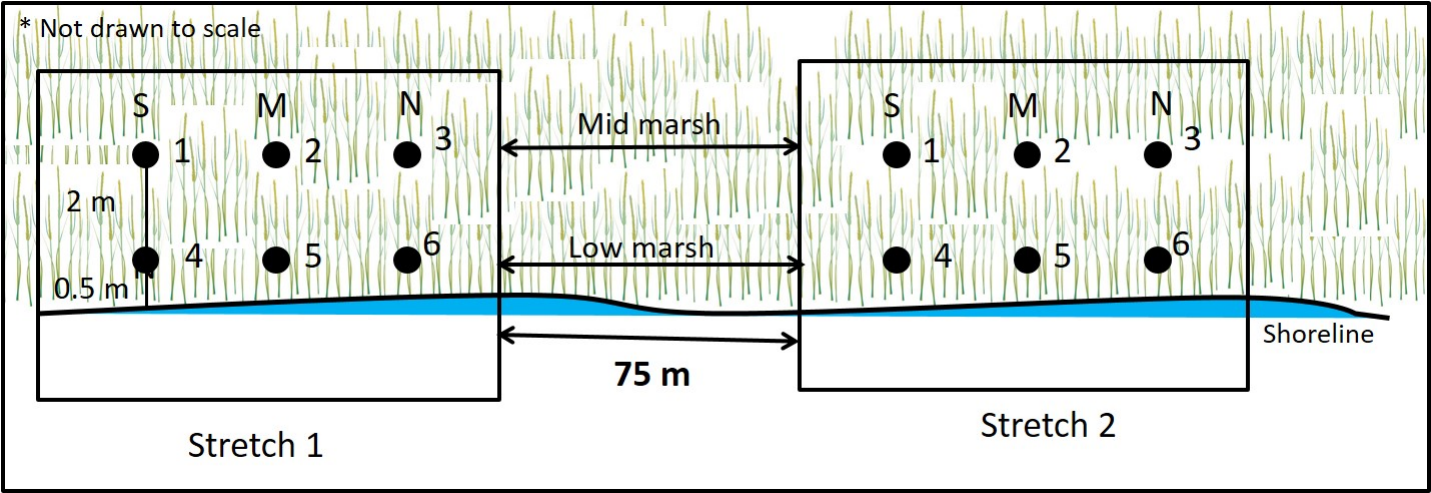
Sampling stations. Stations 1–3 represent mid-marsh stations and stations 4–6 represent low-marsh elevation. S = south, M = mid and N = north stations. (Only stretches 1 and 2 are shown).

Along the shoreline, eight stretches of fringing marsh (numbered 1–8; 1 being the southernmost and 8 being the northernmost; Fig 1B) characterized by varied degree of degradation and bearings to the dominant wind direction were selected. Further, within the selected stretches, high variability of shoreline accretion and erosion was observed. Each stretch of fringing marsh shore measured ca. 75 m linear distance, and was separated by at least 75 m from the adjacent stretches. We were limited in the number of quadrats we could count at the low elevation during high tides. Therefore, we decided to set up permanent quadrats on either side (25 m apart) of the center of each selected shoreline stretch to maximize our chance of obtaining changes in vegetation density representative of the overall shoreline dynamics within the stretch, given the number of quadrats possible. Thus, three parallel replicate transects of length 2.5 m separated by 25 m and running perpendicular to the shoreline were established within each stretch (24 transects in total; Fig 2). The south transect was located about 12.5 m north of the southern stretch limit, and the north transect about 12.5 m south of the northern stretch limit. Fixed sampling stations were established at 0.5 m (low marsh: represented by tall-form *S*. *alterniflora*) and at 2.5 m (mid marsh: represented by short-form *S*. *alterniflora*) landward of the shoreline on transects for a total of 6 sampling stations within each stretch (48 sampling stations in total). Permanent 1-m^2^ quadrats were established at the center of the sampling stations. Within the quadrats, we measured densities of smooth cordgrass (*Spartina alterniflora*). We also analyzed shoreline erosion rates for shoreline stretches using the Digital Shoreline Analysis System (DSAS).

#### Shoreline erosion rate

We measured annual shoreline change rate (recession or advance; expressed as m yr^-1^) by mapping shoreline positon using Global Positioning System (GPS) survey instruments (S1 Table). Shorelines were first mapped in Nov 2008, and then in Nov 2009, Sep 2010 and Jan 2012. We used a Ceeducer Pro differential GPS in 2008, 2009 and 2010, and a Trimble^®^ Real Time Kinematic (RTK) GPS (TSC-2 controller and Trimble-R8 Model-3 rover) in 2012. The shoreline contour, defined as the seaward edge of vegetation, was mapped by holding the Ceeducer Pro DGPS or Trimble RTK GPS receiver in an upright position while walking. Both instruments were programmed to take coordinate measurements (in UTM NAD83 16N) continuously every second, allowing us to create highly accurate maps of the shoreline contour in ESRI ArcGIS 10.0. The seaward edge of vegetation was defined as the seaward-most vegetation band with at least 10 culms per m length of the edge. Positioning error associated with Ceeducer Pro DGPS and Trimble RTK was less than 5 cm.

**Fig 3:**
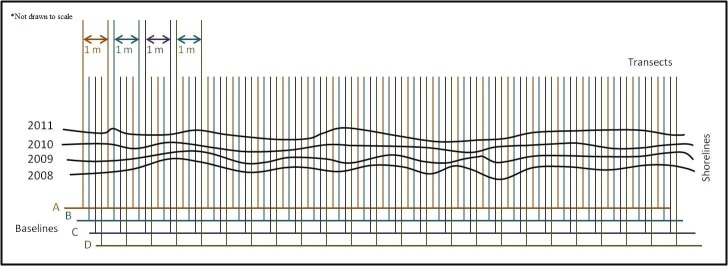
Schematic of shoreline erosion estimation using DSAS technique. (Only subtidal baselines and transects are shown).

Shoreline movement was determined as the annual change in shoreline position (m yr^-1^). For simplicity, we refer the sampling time between 2008–2009 as period I, 2009–2010 as period II, and 2010–2012 as period III (S1 Table). To calculate shoreline position change, we first established eight digital* baselines (i.e. reference lines against which the position of the shoreline was measured), four subtidal and four upland of the shorelines in the DSAS (ver. 4.0) [18]. We then lined up digital* transects at 1-m intervals along each baseline, with transects being perpendicular to baselines. Consecutive transects were offset by ca. 12 cm and, as a result, we generated ca. 600 digital* transects per shoreline stretch. For simplicity, we only show the subtidal transects in Fig 3. Annual changes in shoreline positions for each transect were calculated as the difference in perpendicular distance from the baseline to the shoreline.

All calculations were derived with DSAS, an extension of ArcGIS specifically designed for determining shoreline movement [18]. (* digital here refers to- lines constructed with the computer program. Fig 3 shows only schematic of the shorelines, but not actual diagram of NEPAP shorelines.).

#### *Spartina alterniflora* density

We measured *S*. *alterniflora* density from Jan 2009 to Jan 2012 for a total of 16 sampling events. Corresponding to shoreline mapping events, we determined *S*. *alterniflora* density five times in Jan 2009 to Oct 2009 (period I), six times in Dec 2009 to Aug 2010 (period II) and again five times in Oct 2010 to Jan 2012 (period III; S1 Table). The total number of culms (live and dead) present within the quadrat was counted during low tides when all the culms remained emergent.

#### Statistics

Inter-annual rate of shoreline movement (erosion or advancement) was analyzed using one-way repeated measures ANOVA (RMANOVA) with time (period) as the within-subject factor. In this analysis, we considered ca. 600 digital transects as replicates within each shoreline stretch, and mean shoreline change derived for the stretch as the mean value for all transects in the stretch. The mean values were used as subjects for RMANOVA analysis among time-periods I, II and III; however, in Nov 2008 shoreline data for Stretch #3 was corrupted, therefore only 7 stretches (subjects) were included in RMANOVA analysis. Significant erosion difference, if found among three periods, was followed-up with pairwise post-hoc paired t-test analysis.

Changes in inter-annual *S*. *alterniflora* (total and live) densities were analyzed using one-way RMANOVA with time (period) as the within-subject factor. For these analyses, mean value of each stretch from three replicate stations was used as a subject and compared among periods I, II and III, separately for mid- and low-marsh elevations. Some of the established permanent quadrats were eroded during the survey period due to storm events leading to scarp erosion therefore such quadrats were not included in our analyses. Mean values of the remaining replicate quadrats were used as the subjects for RMANOVA analyses. Quadrats showing zero density values due to gradual population thinning were, however, included in our analyses.

Post-hoc paired t-tests for RMANOVA analyses were done when significant difference was observed among the periods. Data were tested for normality, homoscedasticity, and sphericity and met the conditions for RMANOVA analyses. Analyses were done using SigmaPlot ver. 12.3. All statistical tests were considered significant at α≤0.05 and values are presented as mean ± 1 standard error (SE).

## Results

### Shoreline Dynamics

**Fig 4:**
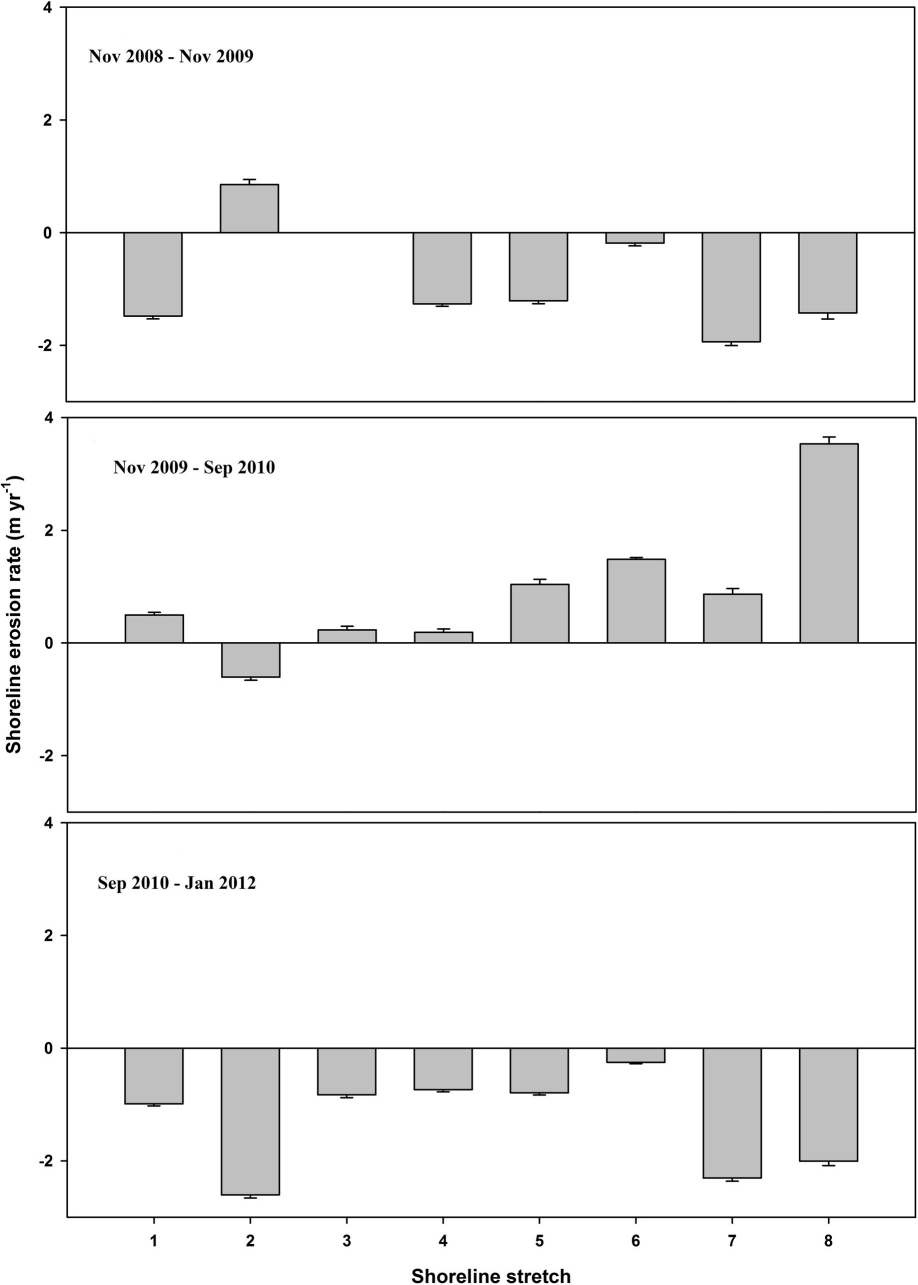
Inter-annual shoreline erosion rate (m yr^-1^). Negative values indicate shoreline erosion; positive values indicate seaward advance.

Shoreline movement showed significant inter-annual variability along the selected stretches (Table 1; Fig 4). Shorelines eroded in period I (mean erosion rate = 1.20 ± 0.02 m yr^-1^) and period III (mean erosion rate = 1.15 ± 0.02 m yr^-1^); whereas, shorelines advanced in period II (mean advancement rate = 1.05 ± 0.02 m yr^-1^) (S2 Table). Despite shoreline advancement in period II, overall shoreline dynamics translated to mean erosion of 0.39 ± 0.01 m yr^-1^ for the entire survey period of Nov 2008 to Jan 2012. Post-hoc pairwise analysis (paired t-test) of shoreline movement followed erosion and accretion pattern (i.e., period II > I = III; Table 1).

**Table 1:** One-way RMANOVA results.

	Time			Residual	Post-hoc tests on time-periods		
	df	F	p	df	I	II	III
Shoreline erosion rate	2	8.466	0.005*	12	A	B	A
*S*. *alterniflora* total density mid marsh	2	12.889	<0.001*	14	A	A	B
*S*. *alterniflora* total density low marsh	2	5.231	0.020*	14	A	AB	B
*S*. *alterniflora* live density mid marsh	2	15.407	<0.001*	14	A	A	B
*S*. *alterniflora* live density low marsh	2	5.312	0.019*	14	A	AB	B

*Asterisks represent significant difference at p<0.05.

### Permanent quadrats

Out of 48 established quadrats, seven quadrats were lost owing to undercutting, collapse or scarp erosion (i.e., loss of whole marsh mass). Scarp erosion was clearly visible during each visit at the study site, especially along the northern ridges of Point-aux-Pins shoreline (i.e., stretches 5–7). Such eroded quadrats were not included in our analyses. Eroded quadrats never revegetated and eventually degraded into bare sediment flats. Table 2 summarizes the number of quadrats lost at each stretch during different periods.

**Table 2:** Number of permanent quadrats lost at each shoreline stretch.

Stretch	No. of quadrats lost	Marsh position	Period
#1	1	Low	III
#3	1	Low	III
#5	1	Mid	III
#5	1	Low	II
#5	1	Low	III
#7	1	Low	I
#7	1	Low	II
Total	7	Low-6; Mid-1	I-1; II-2; III-4

### *Spartina alterniflora* density

**Fig 5:**
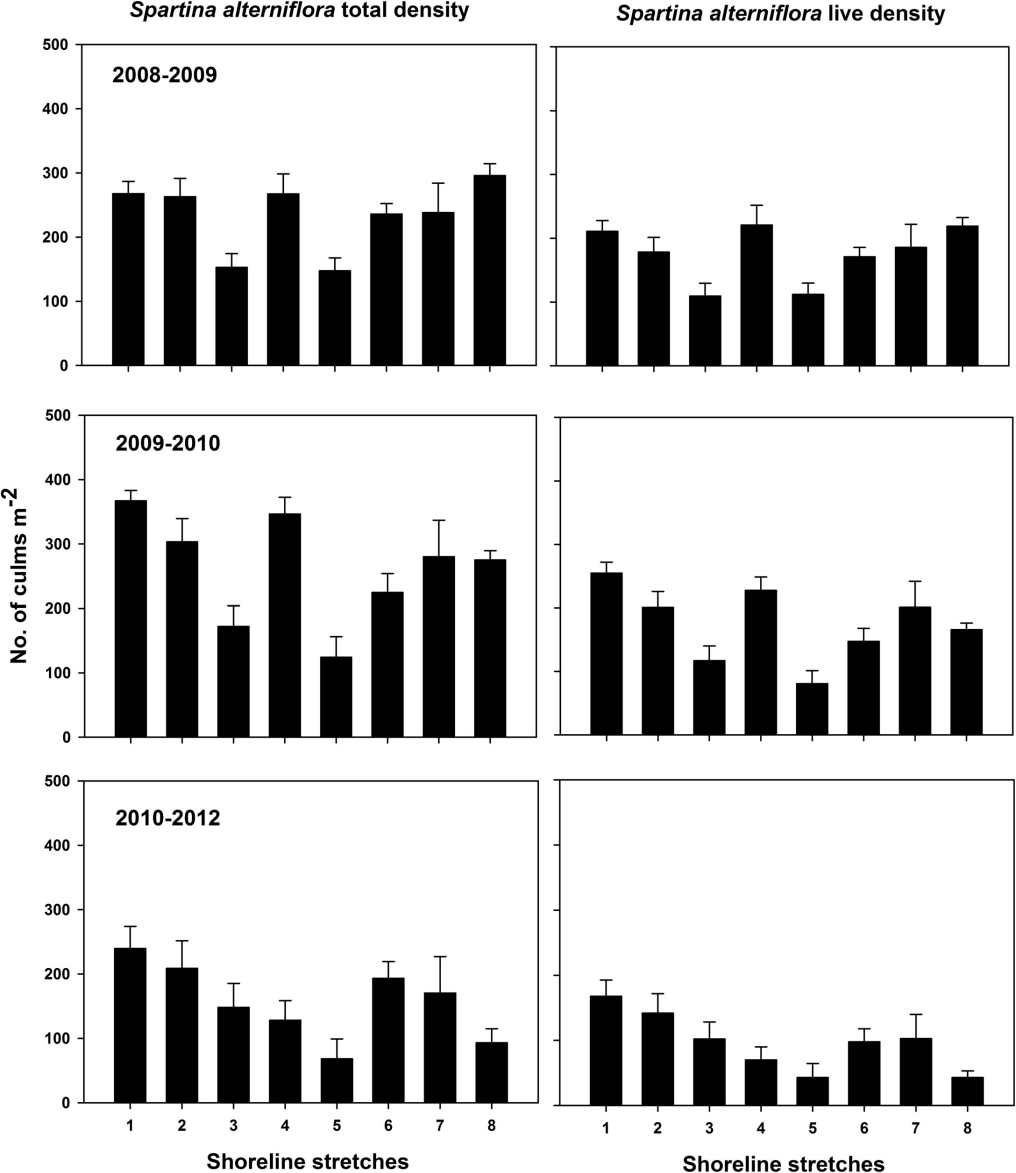
Mid-marsh total and live *S*. *alterniflora* density. Error bars ±1 SE.

**Fig 6:**
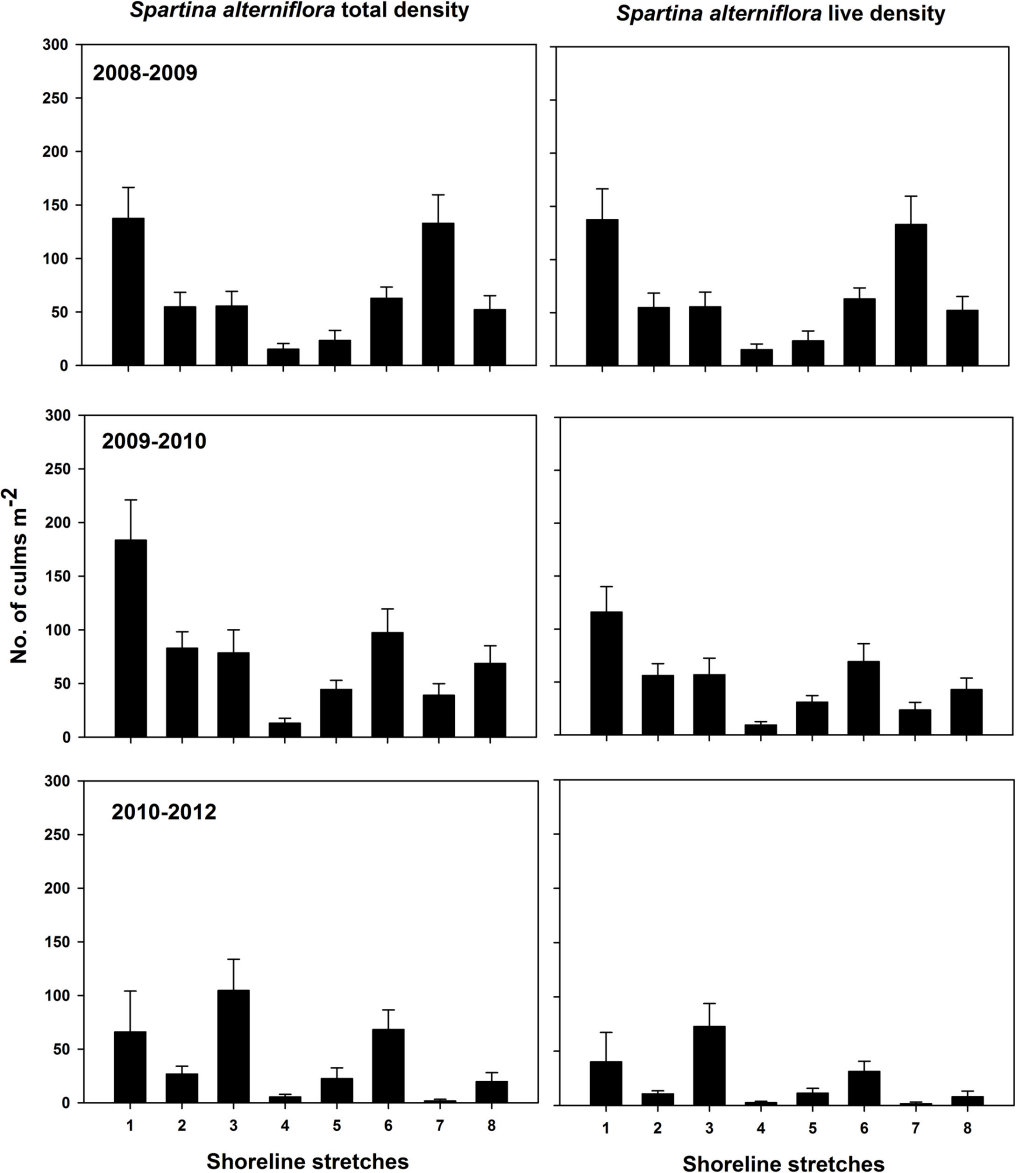
Low-marsh total and live *S*. *alterniflora* density. Error bars ±1 SE.

Total (live and dead) *S*. *alterniflora* density ranged 5–606 stems m^-2^ (mean = 221.1 ± 7.5) for mid-marsh plots (S3 Table; Fig 5) and 5–504 stems m^-2^ (mean = 77.3 ± 5.2) for low-marsh plots (S4 Table; Fig 6). Live *S*. *alterniflora* density ranged 1–450 stems m^-2^ (mean = 151.1 ± 5.6) for mid-marsh plots (S5 Table; Fig 5) and 4–399 stems m^-2^ (mean = 49.5 ± 3.6) for low-marsh plots (S6 Table; Fig 6). On average, at mid elevation live culms represented 73, 66, and 57% of total culms enumerated for periods I, II and III respectively, whereas for low marsh the live culm composition was 61, 65 and 41% for periods I, II and III respectively.

Significant differences in total and live *S*. *alternilfora* density among periods were observed for both elevations (Table 1). Total and live *S*. *alterniflora* density followed identical post-hoc paired t-test analyses revealing period I = II > III (for mid marsh) and period I = II, II = III but I ≠ III (for low-marsh; Table 1).

Based on our analyses, two salient results of marsh vegetation were observed. First, there was a gradual decline in vegetation density from period I to III and more abrupt decline was observed in low elevation marshes than mid elevation marshes (Figs 5 and 6). Second, despite shoreline advancement in period II, no corresponding and consistent increase in vegetation density was observed (Figs 4–6).

## Discussion

Among numerous disturbances, lateral erosion is probably most persistent along the Gulf coast [19], and unlike other disturbances (such as wrack deposition and extended flooding period), erosion can potentially result in loss of marsh area, and quality [16]. Although, scientific literature is abound with increased concerns about potential sea level rise and its consequent deleterious impacts on coastal wetlands, lateral erosion, may have much direct and larger effects on these systems [20]. Fringing marshes, due to their proximity with marine disturbances, are the first vegetation to respond to shoreline erosion; therefore, we chose marsh fringes to study the response of shoreline dynamics (lateral shoreline movement) on marsh vegetation.

We found highly variable shoreline positon along the selected stretches at our study site. Almost all stretches (except stretch #2) showed erosion during the first year of survey; however, the trend was reversed the following year where shoreline advancement was observed in most stretches (except stretch #2). All shoreline stretches showed erosion in the third year of survey. Although we surveyed shorelines in the fall for Periods I (Nov) and III (Jan) and late summer (Sep) in Period II, we do not consider this as the underlying reason for shoreline dynamics among the three periods because in the southern USA, the growing season is longer in comparison to the Atlantic coasts and fall senescence is slower. Additionally, many young culms originated in the fall live through the winter. Culms originated in the previous spring do not completely senescence until the end of winter [21]. NEPAP shorelines are likely subjected to impulses of inter-annual wind and current regimes, which largely determine local annual shoreline oscillations. Although shorelines advanced in period II, an overall net erosion was observed for the survey period. Indeed, NEPAP and surrounding areas, in the absence of any protective mechanisms, have been experiencing chronic erosion in the region since 1950s or even before [22].

A general decline in vegetation density also was noticed throughout the survey period. Two distinct mechanisms of marsh loss were observed at NEPAP. First, especially along the northern shorelines, large areas of marsh were eroded due to scarp edge failure, thus resulting in loss of intact marsh sediment with above- and below-ground vegetation. This kind of marsh loss has been referred to as “cliff erosion” in the literature, and is mostly associated with high-energy events. Steep escarpments are characteristic features of cliff erosion, and some escarpments were detected along the northern shorelines, indicting high energy at the northern bend of NEPAP [15, 16]. Indeed, the northern shoreline of NEPAP is perpendicular to dominant wind direction, thus partly explaining associated high energy and resulting escarpments. Under favorable conditions, even scarped marsh failure can colonize new sediment flats; however, we did not observe new colonization by eroded marsh blocks indicating relatively high energy in the area [15]. Second, we noticed gradual density decline, sometimes leading to zero density values. This kind of response shown by biota likely was due to a result of cumulative stress exerted by long-term lateral disturbances. Southern stretches of NEPAP shores mostly exhibited population thinning. We also noticed that decline in vegetation density at low elevation was more abrupt than mid elevation, likely due to their immediate proximity to erosive wave processes.

**Fig 7:**
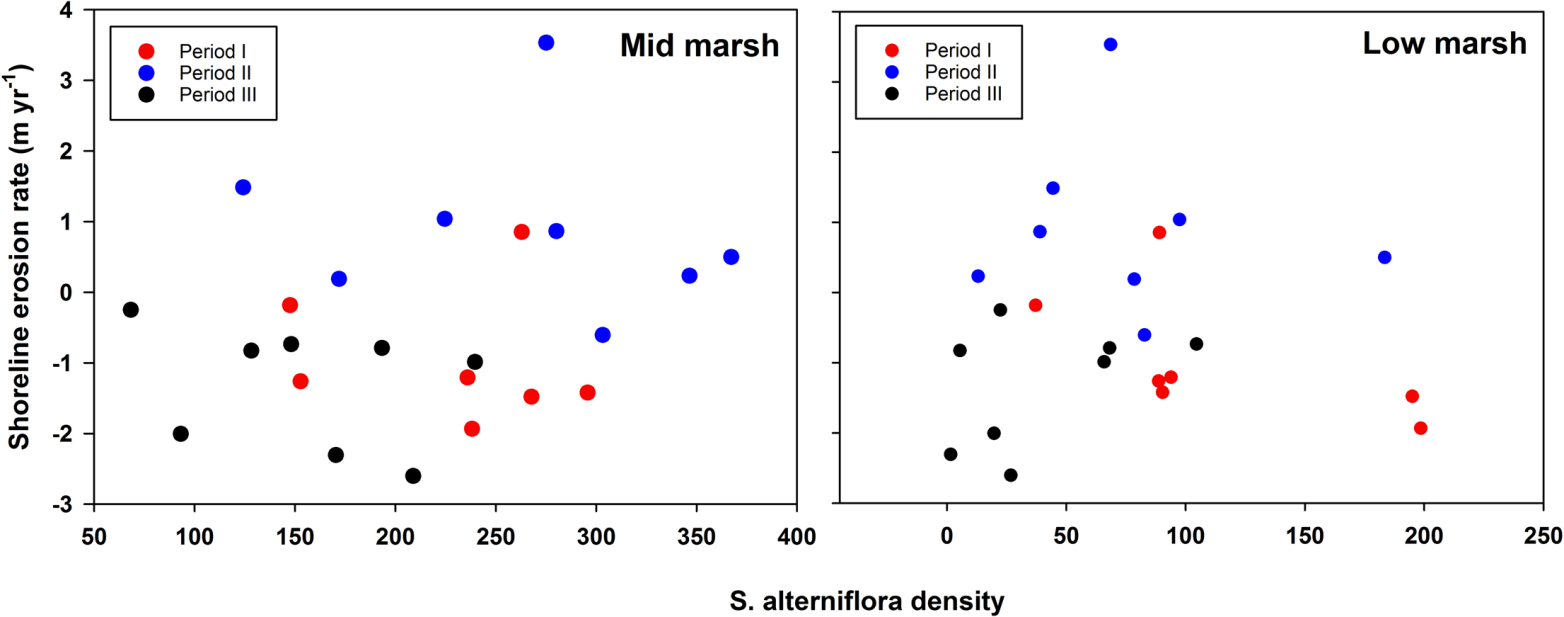
Scatterplot of S. alterniflora vs. Shoreline erosion rate for Mid- and Low-marsh.

Although shorelines advanced in year 2, marsh edge did not display corresponding increase in density. This suggests that marsh biota may require a longer time to recover from shoreline erosion. Further, in the following year (period III) shorelines again showed recession, which did not allow adequate time for newly recruited biota, if any, to respond to a brief shoreline advancement in the previous year. Our analyses revealed that shoreline erosion and marsh vegetation density do not follow one another in tandem, yet, a general decline in vegetation density as well as exacerbated shoreline erosion was observed in year 3 (Fig 7). *S*. *alterniflora* density values among different periods plotted against shoreline erosion rate (m yr^-1^) did not show any significant trend (Fig 7). If density values were contingent on shoreline dynamics, some specific trend should have emerged, but evidently that was not the case. We admit that there were some caveats in distribution of our sampling times within different periods. For period I, the timeline was well balanced among different seasons. However, later periods (II and III) did not have equal sampling distribution among different seasons. In period II, three out of six sampling dates belonged to winter and in period III, three out of five sapling dates belonged to the fall season. To overcome this potential bias, we conducted an identical set of analyses but only encompassing data from *S*. *alterniflora* growing season (i.e., from March to September). However, we obtained statistically similar results for both elevations, as shown in Table 1. Study period encompassing more sampling rounds could have helped in determining the trend of vegetation density extending beyond our three-year study period.

Finally, we observed a general decrease in percent composition of live culms from periods I to III. Percent decrease in live culms through periods I to III could be attributed to disparity in sampling season (i.e., periods II and III had more samples from non-growing season of cordgrass); however, it may also have been due to progressive stress of lateral erosion in each period leading to lower percent composition of live culms in the following period. Although there was a general decrease in marsh vegetation at our study area, it can also be acknowledged that antecedent plant density had been unable to resist lateral erosion. Overall, low density of the antecedent marsh edge, coupled with relatively high wave energy in the study area, may have worked in tandem to aggravate vegetation condition. Elsewhere, *Spartina alterniflora* density has been reported in much higher values, for example, densities of 300–400 stems m^-2^ on Dauphin Island, Alabama [23], 130–222 in Outer Banks, North Carolina [24], 516–890 in coastal South Carolina [25], and 132–469 in coastal Virginia [26] have been reported. Higher fringing marsh density could have resisted lateral disturbances more effectively but we were unable to empirically test this condition. More research related to marsh density and shoreline change is expected to assist resource managers in developing management strategies for coastal marshes against lateral disturbances.

Based on our results, we recommend to the coastal resources planners that low-elevation marshes are the first responders of lateral disturbances arising from the seaward side, therefore low-elevation marshes should be prioritized for protection. If the protection strategy incorporates marsh planting, higher density should be aimed towards the lower elevation. More structural complexity associated with higher vegetation density in turn aids marshes in combating against lateral disturbances due to increased lateral sediment trapping and wave attenuation. Further, positive consequences (increase in culm density) may cascade to mid- and high-elevation marshes as well. The exact marsh density to be restored as a means of protective or restorative strategy depends on an array of factors such as salinity, inundation period, wave energy, sediment quality etc. Protective strategies incorporating “living shoreline” approaches have revealed positive results along the Gulf coasts and beyond. Lately, hybrid designs (i.e., an integration of biogenic resources with engineering structures) have also been considered to be highly efficacious in erosion control and marsh stabilization, and specifically, in coastal Alabama, such hybrid designs have shown some promising prospects [22].

## Conclusions

In conclusion, our study determined the impact of lateral shoreline dynamics on fringing marsh density. Shorelines showed high oscillations during the survey period, with recession in year 1 and year 3, but advance in year 2. Despite shoreline advance in year 2, overall net erosion was observed for the selected shoreline stretches. Fringing *S*. *alterniflora* density did not follow trends in shoreline erosion; however, an overall decrease in marsh density was observed. Low elevation marsh showed more abrupt decline in density than mid-elevation marsh likely due to their proximity to destructive wave processes. These results are expected to benefit the design of protective plans for coastal systems against lateral erosion.

## Supporting Information

S1 Table Sampling dates. (DOCX)

S2 Table Shoreline erosion. (DOCX)

S3 Table Total density mid marsh. (DOCX)

S4 Table Live density mid marsh. (DOCX)

S5 Table Total density low marsh. (DOCX)

S6 Table Live density low marsh. (DOCX)
