# Supplementary material for: Effects of Shoreline Dynamics on Saltmarsh Vegetation
Source: PLoS One. 2016 Jul 21;11(7):e0159814. doi: 10.1371/journal.pone.0159814 (PMC4956348; doi:10.1371/journal.pone.0159814)
Supplement: S1 Table — (DOCX) [file pone.0159814.s001.docx]

**S1 Table: Sampling Date**

| Period | Metrics |  |
| --- | --- | --- |
|  | Shoreline mapping | *S. alterniflora* density |
| I | Nov-08 |  |
| I |  | 1/9/2009 |
| I |  | 5/26/2009 |
| I |  | 6/22/2009 |
| I |  | 7/20/2009 |
| I |  | 10/19/2009 |
|  |  |  |
| II | Nov-09 |  |
| II |  | 12/4/2009 |
| II |  | 1/12/2010 |
| II |  | 2/10/2010 |
| II |  | 4/20/2010 |
| II |  | 6/23/2010 |
| II |  | 8/6/2010 |
|  |  |  |
| III | Sep-10 |  |
| III |  | 10/29/2010 |
| III |  | 3/1/2011 |
| III |  | 9/20/2011 |
| III |  | 11/16/2011 |
|  | Jan-12 | 1/31/2012 |
