# Supplementary material for: Effects of Shoreline Dynamics on Saltmarsh Vegetation
Source: PLoS One. 2016 Jul 21;11(7):e0159814. doi: 10.1371/journal.pone.0159814 (PMC4956348; doi:10.1371/journal.pone.0159814)
Supplement: S2 Table — (DOCX) [file pone.0159814.s002.docx]

**S2 Table: Shoreline erosion**

| Year | Period | Stretch | Mean erosion rate | Standard Error | No. of transects |
| --- | --- | --- | --- | --- | --- |
| 2008-2009 | I | 1 | -1.48059 | 0.049658 | 612 |
| 2008-2009 | I | 2 | 0.85325 | 0.091239 | 84 |
| 2008-2009 | I | 4 | -1.26346 | 0.04201 | 560 |
| 2008-2009 | I | 3 |  |  |  |
| 2008-2009 | I | 6 | -0.18481 | 0.049203 | 560 |
| 2008-2009 | I | 5 | -1.20843 | 0.051924 | 612 |
| 2008-2009 | I | 7 | -1.93449 | 0.067616 | 584 |
| 2008-2009 | I | 8 | -1.42331 | 0.109187 | 560 |
| 2009-2010 | II | 1 | 0.49991 | 0.044005 | 612 |
| 2009-2010 | II | 2 | -0.60652 | 0.056797 | 84 |
| 2009-2010 | II | 4 | 0.18725 | 0.061719 | 560 |
| 2009-2010 | II | 3 | 0.2321 | 0.064483 | 644 |
| 2009-2010 | II | 6 | 1.48534 | 0.032722 | 560 |
| 2009-2010 | II | 5 | 1.0394 | 0.091166 | 612 |
| 2009-2010 | II | 7 | 0.86589 | 0.103167 | 584 |
| 2009-2010 | II | 8 | 3.53279 | 0.122802 | 560 |
| 2010-2011 | III | 1 | -0.98972 | 0.033619 | 612 |
| 2010-2011 | III | 2 | -2.60384 | 0.05741 | 84 |
| 2010-2011 | III | 4 | -0.73635 | 0.039058 | 560 |
| 2010-2011 | III | 3 | -0.82875 | 0.048961 | 644 |
| 2010-2011 | III | 6 | -0.25121 | 0.02528 | 560 |
| 2010-2011 | III | 5 | -0.79159 | 0.041252 | 612 |
| 2010-2011 | III | 7 | -2.30603 | 0.05657 | 584 |
| 2010-2011 | III | 8 | -2.00723 | 0.076452 | 560 |
