# Supplementary material for: Effects of Shoreline Dynamics on Saltmarsh Vegetation
Source: PLoS One. 2016 Jul 21;11(7):e0159814. doi: 10.1371/journal.pone.0159814 (PMC4956348; doi:10.1371/journal.pone.0159814)
Supplement: S3 Table — (DOCX) [file pone.0159814.s003.docx]

**S3 Table: Total Density Mid Marsh**

| Period | Stretch | Position | Total Density | Standard Error | No. of transects |
| --- | --- | --- | --- | --- | --- |
| I | 1 | Mid | 267.733 | 18.8039 | 15 |
| I | 2 | Mid | 262.933 | 28.6715 | 15 |
| I | 3 | Mid | 152.8 | 21.4057 | 15 |
| I | 4 | Mid | 267.4 | 30.9751 | 15 |
| I | 5 | Mid | 147.533 | 20.1508 | 15 |
| I | 6 | Mid | 235.933 | 16.2675 | 15 |
| I | 7 | Mid | 238.2 | 45.9959 | 15 |
| I | 8 | Mid | 295.733 | 18.7481 | 15 |
| II | 1 | Mid | 367.222 | 16.0111 | 18 |
| II | 2 | Mid | 303.222 | 36.299 | 18 |
| II | 3 | Mid | 171.944 | 32.2565 | 18 |
| II | 4 | Mid | 346.5 | 26.0515 | 18 |
| II | 5 | Mid | 124.167 | 31.966 | 18 |
| II | 6 | Mid | 224.556 | 29.315 | 18 |
| II | 7 | Mid | 280.167 | 56.679 | 18 |
| II | 8 | Mid | 275.111 | 14.5739 | 18 |
| III | 1 | Mid | 239.667 | 34.2049 | 15 |
| III | 2 | Mid | 208.867 | 42.8696 | 15 |
| III | 3 | Mid | 148.133 | 37.1956 | 15 |
| III | 4 | Mid | 128.267 | 30.3441 | 15 |
| III | 5 | Mid | 68.25 | 30.8246 | 12 |
| III | 6 | Mid | 193.333 | 26.0318 | 15 |
| III | 7 | Mid | 170.4 | 56.8032 | 15 |
| III | 8 | Mid | 93.133 | 21.8553 | 15 |
