# Supplementary material for: Effects of Shoreline Dynamics on Saltmarsh Vegetation
Source: PLoS One. 2016 Jul 21;11(7):e0159814. doi: 10.1371/journal.pone.0159814 (PMC4956348; doi:10.1371/journal.pone.0159814)
Supplement: S4 Table — (DOCX) [file pone.0159814.s004.docx]

**S4 Table: Total Density Mid Marsh**

| Period | Stretch | Position | Live density | Standard Error | No. of transects |
| --- | --- | --- | --- | --- | --- |
| I | 1 | Mid | 210.8 | 16.9369 | 15 |
| I | 2 | Mid | 178.333 | 23.1038 | 15 |
| I | 3 | Mid | 109.533 | 19.9626 | 15 |
| I | 4 | Mid | 220.733 | 30.882 | 15 |
| I | 5 | Mid | 111.933 | 17.7945 | 15 |
| I | 6 | Mid | 170.867 | 15.0019 | 15 |
| I | 7 | Mid | 185.733 | 36.3714 | 15 |
| I | 8 | Mid | 218.933 | 13.6303 | 15 |
| II | 1 | Mid | 254.5 | 17.3402 | 18 |
| II | 2 | Mid | 200.833 | 24.6927 | 18 |
| II | 3 | Mid | 116.833 | 23.2571 | 18 |
| II | 4 | Mid | 227.333 | 21.2481 | 18 |
| II | 5 | Mid | 80.833 | 20.4802 | 18 |
| II | 6 | Mid | 147.278 | 20.6845 | 18 |
| II | 7 | Mid | 201.111 | 40.7274 | 18 |
| II | 8 | Mid | 165.722 | 10.3667 | 18 |
| III | 1 | Mid | 167.6 | 24.9693 | 15 |
| III | 2 | Mid | 141.667 | 30.1365 | 15 |
| III | 3 | Mid | 102.067 | 26.1724 | 15 |
| III | 4 | Mid | 70.067 | 19.8649 | 15 |
| III | 5 | Mid | 42.833 | 21.5866 | 12 |
| III | 6 | Mid | 97.867 | 19.9907 | 15 |
| III | 7 | Mid | 102.533 | 37.2386 | 15 |
| III | 8 | Mid | 42.867 | 10.2395 | 15 |
