# Supplementary material for: Effects of Shoreline Dynamics on Saltmarsh Vegetation
Source: PLoS One. 2016 Jul 21;11(7):e0159814. doi: 10.1371/journal.pone.0159814 (PMC4956348; doi:10.1371/journal.pone.0159814)
Supplement: S5 Table — (DOCX) [file pone.0159814.s005.docx]

**S5 Table: Total Density Low Marsh**

| Period | Stretch | Position | Total Density | Standard Error | No. of transects |
| --- | --- | --- | --- | --- | --- |
| I | 1 | Low | 195.067 | 34.2159 | 15 |
| I | 2 | Low | 89.067 | 14.9132 | 15 |
| I | 3 | Low | 88.733 | 19.2372 | 15 |
| I | 4 | Low | 34.2 | 9.8634 | 15 |
| I | 5 | Low | 37.133 | 13.4387 | 15 |
| I | 6 | Low | 93.933 | 14.6617 | 15 |
| I | 7 | Low | 198.6 | 40.4077 | 15 |
| I | 8 | Low | 90.4 | 21.9961 | 15 |
| II | 1 | Low | 183.5 | 37.6256 | 18 |
| II | 2 | Low | 82.833 | 15.4825 | 18 |
| II | 3 | Low | 78.5 | 21.5951 | 18 |
| II | 4 | Low | 13.056 | 4.5009 | 18 |
| II | 5 | Low | 44.462 | 8.531 | 13 |
| II | 6 | Low | 97.5 | 22.0397 | 18 |
| II | 7 | Low | 39 | 10.9575 | 10 |
| II | 8 | Low | 68.611 | 16.5802 | 18 |
| III | 1 | Low | 65.917 | 38.1981 | 12 |
| III | 2 | Low | 26.8 | 7.2691 | 15 |
| III | 3 | Low | 104.583 | 29.1133 | 12 |
| III | 4 | Low | 5.467 | 2.3762 | 15 |
| III | 5 | Low | 22.429 | 10.0827 | 7 |
| III | 6 | Low | 68.2 | 18.3535 | 15 |
| III | 7 | Low | 1.6 | 1.6 | 5 |
| III | 8 | Low | 19.733 | 8.3384 | 15 |
