# Supplementary material for: Effects of Shoreline Dynamics on Saltmarsh Vegetation
Source: PLoS One. 2016 Jul 21;11(7):e0159814. doi: 10.1371/journal.pone.0159814 (PMC4956348; doi:10.1371/journal.pone.0159814)
Supplement: S6 Table — (DOCX) [file pone.0159814.s006.docx]

**S6 Table: Live Density Low Marsh**

| Period | Stretch | Position | Live Density | Standard Error | No. of Transects |
| --- | --- | --- | --- | --- | --- |
| I | 1 | Low | 137.467 | 28.949 | 15 |
| I | 2 | Low | 54.8 | 13.6675 | 15 |
| I | 3 | Low | 55.6 | 13.8071 | 15 |
| I | 4 | Low | 15.267 | 5.343 | 15 |
| I | 5 | Low | 23.467 | 9.364 | 15 |
| I | 6 | Low | 62.867 | 10.4639 | 15 |
| I | 7 | Low | 132.933 | 26.6636 | 15 |
| I | 8 | Low | 52.2 | 13.0968 | 15 |
| II | 1 | Low | 116.056 | 23.8417 | 18 |
| II | 2 | Low | 56.111 | 11.2502 | 18 |
| II | 3 | Low | 56.833 | 15.7553 | 18 |
| II | 4 | Low | 9.333 | 3.2186 | 18 |
| II | 5 | Low | 30.923 | 6.3351 | 13 |
| II | 6 | Low | 69.222 | 17.0984 | 18 |
| II | 7 | Low | 23.5 | 7.253 | 10 |
| II | 8 | Low | 42.833 | 10.9244 | 18 |
| III | 1 | Low | 40.083 | 27.161 | 12 |
| III | 2 | Low | 10.667 | 2.8093 | 15 |
| III | 3 | Low | 72.667 | 21.2087 | 12 |
| III | 4 | Low | 2.6 | 1.1904 | 15 |
| III | 5 | Low | 11.286 | 4.5025 | 7 |
| III | 6 | Low | 31.4 | 9.3716 | 15 |
| III | 7 | Low | 1.6 | 1.6 | 5 |
| III | 8 | Low | 8 | 5.2681 | 15 |
